# Is immunosuppression status a risk factor for noninvasive ventilation failure in patients with acute hypoxemic respiratory failure? A post hoc matched analysis

**DOI:** 10.1186/s13613-019-0566-z

**Published:** 2019-08-14

**Authors:** Rémi Coudroy, Tài Pham, Florence Boissier, René Robert, Jean-Pierre Frat, Arnaud W. Thille

**Affiliations:** 1 Service de Médecine Intensive et Réanimation, CHU de Poitiers, Poitiers, France; 2 INSERM CIC 1402, ALIVE, Université de Poitiers, Poitiers, France; 3 Interdepartmental Division of Critical Care, University of Toronto, Toronto, Canada; 4 Keenan Research Center and Li Ka Shing Knowledge Institute, Toronto, Canada

**Keywords:** Immunosuppression, Acute respiratory distress syndrome, Noninvasive ventilation

## Abstract

**Background:**

Recent European/American guidelines recommend noninvasive ventilation (NIV) as a first-line therapy to manage acute hypoxemic respiratory failure in immunocompromised patients. By contrast, NIV may have deleterious effects in nonimmunocompromised patients and experts have been unable to offer a recommendation. Immunocompromised patients have particularly high mortality rates when they require intubation. However, it is not clear whether immunosuppression status is a risk factor for NIV failure. We assessed the impact of immunosuppression status on NIV failure in a post hoc analysis pooling two studies including patients with de novo acute hypoxemic respiratory failure treated with NIV. Patients with hypercapnia, acute exacerbation of chronic lung disease, cardiogenic pulmonary edema, or with do-not-intubate order were excluded.

**Results:**

Among the 208 patients included in the analysis, 71 (34%) were immunocompromised. They had higher severity scores upon ICU admission, higher pressure-support levels, and minute ventilation under NIV, and were more likely to have bilateral lung infiltrates than nonimmunocompromised patients. Intubation and in-ICU mortality rates were higher in immunocompromised than in nonimmunocompromised patients: 61% vs. 43% (*p* = 0.02) and 38% vs. 15% (*p* < 0.001), respectively. After adjustment or using a propensity score-matched analysis, immunosuppression was not associated with intubation, whereas it remained independently associated with ICU mortality with an adjusted odds ratio of 2.64 (95% CI 1.24–5.67, *p* = 0.01).

**Conclusions:**

Immunosuppression status may directly influence mortality but does not seem to be associated with an increased risk of intubation in patients with de novo acute hypoxemic respiratory failure treated with NIV. Studies in this specific population are needed.

**Electronic supplementary material:**

The online version of this article (10.1186/s13613-019-0566-z) contains supplementary material, which is available to authorized users.

## Introduction

Application of noninvasive ventilation (NIV) remains debated in patients admitted to intensive care unit (ICU) for de novo acute hypoxemic respiratory failure [1–13], and clinical practice guidelines remain unable to offer a recommendation given the uncertainty of evidence [14]. By contrast, the guidelines suggest NIV as first-line therapy for oxygenation in immunocompromised patients [14]. Indeed, by pooling all randomized controlled trials, NIV has been associated with lower intubation and mortality rates than standard oxygen therapy [15–18].

Mortality of immunocompromised patients requiring intubation and invasive mechanical ventilation is particularly high, ranging from 50 to 70% [19, 20]. Therefore, oxygenation strategies aiming at preventing intubation of these patients deserve consideration. Although recommendations for the management of acute respiratory failure differ between immunocompromised and nonimmunocompromised patients, it is not clear whether or not immunocompromised patients are actually at increased risk of intubation. A recent study including a large sample of patients with acute respiratory distress syndrome (ARDS) reported higher intubation rates in immunocompromised patients than in the others [21]. However, immunocompromised patients had more severe organ failure than nonimmunocompromised patients, and based on unadjusted analysis, this result may simply indicate greater disease severity.

The aim of our study was to assess whether immunosuppression is an actual risk factor for NIV failure by performing post hoc analysis of two studies including immunocompromised and nonimmunocompromised patients admitted to ICU for de novo acute hypoxemic respiratory failure and treated with NIV. The primary aim was to compare adjusted intubation rates according to immunosuppression status in the matched cohort. The secondary aim was to compare in-ICU mortality rates according to immunosuppression status in the matched cohort.

## Methods

### Patients

Patients from a multicenter randomized controlled trial and a retrospective monocenter analysis of prospectively collected data in a high NIV case volume center sharing the same inclusion criteria were included in the present analysis [11, 22]. All patients were admitted to ICU for de novo acute hypoxemic respiratory failure and were treated with NIV as first-line ventilatory support. Acute hypoxemic respiratory failure was defined by a respiratory rate of at least 25 breaths/min and/or clinical signs of respiratory distress with PaO_2_/FiO_2_ ≤ 300 mmHg. De novo respiratory failure implies that patients with acute exacerbation of chronic lung disease or cardiogenic pulmonary edema were excluded. Patients with hypercapnia (PaCO_2_ > 45 mmHg) were also excluded in both studies. For the current analysis, patients who were not treated with NIV (namely those randomized in the standard oxygen group or the high-flow nasal cannula group in the trial from Frat and colleagues [11]), those who were immediately intubated, and those who had a do-not-intubate order were excluded. As a consequence, all nonsurvivors were previously intubated after NIV failure. All included patients were treated in ICU by 1-h to 2-h intermittent sessions of NIV interspaced with either standard oxygen or high-flow nasal cannula oxygen therapy until improvement or intubation. This study was reviewed by our local Institutional Review Board and exempted from requiring approval.

### Data collection

Demographic data such as age, gender, immunosuppression status [23], the main cause of acute respiratory failure, and severity according to simplified acute physiology score II (SAPS II) were collected [24]. Risk factors for acute respiratory failure were collected according to the ARDS risk factors classification from Ferguson and colleagues [25]. Bilateral lung infiltrates were assessed on a chest imaging. Vital signs, oxygen flow, and arterial blood gases under standard oxygen therapy were collected. Vital signs, ventilator settings including FiO_2_, pressure-support, positive end-expiratory pressure (PEEP) level, expired tidal volume (Vte), and arterial blood gas analyses were collected 1 h after NIV initiation as previously reported [26]. The worst PaO_2_/FiO_2_ within the first 24 h after ICU admission under NIV and criteria for ARDS was assessed under NIV according to the Berlin definition [27].

### Statistical analysis

Continuous data were expressed as median [25th–75th percentile] and were compared using the Mann–Whitney or Student *t* test as appropriate. Categorical variables were expressed as percentage and compared using the Chi-square test or Fisher’s exact test as appropriate. Kaplan–Meier curves were plotted to compare time from NIV initiation to intubation or mortality in the ICU by means of the log-rank test. Variables associated with NIV failure or mortality with *p* value < 0.15 using univariate analysis were entered in a stepwise backward logistic regression model to identify noncollinear early variables independently associated with outcomes. Despite its association with poor outcome [24], SAPS II was not entered in the multivariate model given the risk of collinearity with immunosuppression status. Indeed, neutropenia, hematologic cancer, and metastatic cancer increase SAPS II [24]. Moreover, SAPS II cannot be used to predict intubation as it is calculated 24 h after ICU admission, whereas the majority of intubated patients for acute respiratory failure need intubation within the first 24 h. A propensity score was computed according to baseline imbalances between groups, known risk factors for intubation [27], and results from multivariate analyses. Patients were matched according to the nearest score with a 1:1 ratio using a caliper of 0.25 SD and compared using the Wilcoxon signed-rank test, the paired Student *t* test, or the MacNemar test, as appropriate. As in the overall population, Kaplan–Meier survival curves were plotted and compared using the log-rank test, and multivariate analyses were performed to identify variables associated with outcomes in the matched cohort. Two-tailed *p* values < 0.05 were considered significant. Statistical analysis was conducted using R software (available online at http://www.R-project.org).

## Results

**Fig. 1:**
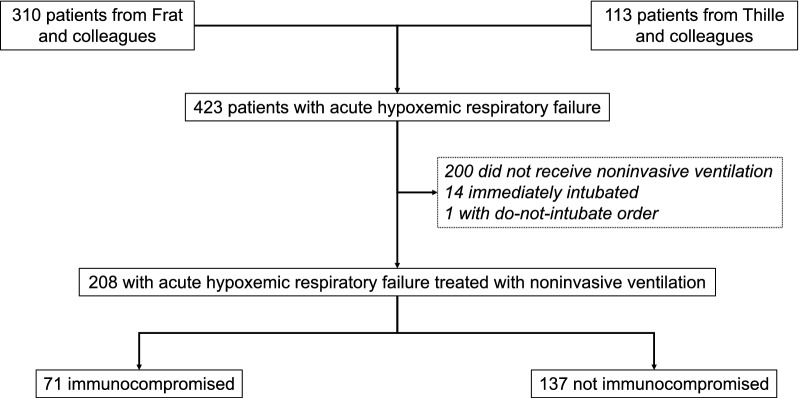
Flow chart of patients included in the study

From the two studies, 423 patients were admitted to ICU for de novo acute hypoxemic respiratory failure. Among the 223 treated with NIV, 208 were retained in the analysis including 71 immunocompromised patients (34%) (Fig. 1). The reasons for immunosuppression were hematologic cancer in 28 cases (39%), immunosuppressive drugs for connective tissue diseases in 13 (18%), solid organ transplantation in 11 (15%), acquired immunodeficiency syndrome in 10 (14%), and solid cancer in 9 (12%). Eleven patients (15%) had undergone allogenic stem cell transplantation, and 9 (13%) had neutropenia. The etiology of acute respiratory failure was pneumonia in 55 cases (77%), specific pulmonary infiltration in 4 (5.6%), extrapulmonary infection in 3 (4.2%), toxic in 3 (4.2%), and intra-alveolar hemorrhage in 3 (4.2%), whereas the 3 remaining patients (4.2%) had no etiological diagnosis for respiratory failure. In the nonimmunocompromised group, 114 patients (83%) had no comorbidities, 7 (5.2%) had coronary insufficiency, 5 (3.6%) had central neurological disorder, 4 (2.9%) had chronic heart failure, 3 (2.2%) had sickle cell disease, and 2 (1.5%) had chronic kidney failure and cirrhosis. Their cause of respiratory failure was pneumonia in 102 cases (74%), extrapulmonary infection in 13 (9.5%), toxic in 4 (2.9%), and miscellaneous in 18 cases (13%).

### Comparison of immunocompromised versus nonimmunocompromised patients in the overall population

Immunocompromised patients had higher SAPS II, and higher pressure-support levels and minute ventilation 1 h after NIV initiation than nonimmunocompromised patients (Table 1). They were more likely than nonimmunocompromised patients to have bilateral lung infiltrates and to meet the criteria for ARDS under NIV (Table 1). Their crude intubation and mortality rates were higher than in nonimmunocompromised patients using univariate analysis or log-rank test (Table 1, Additional file 1: Figure S1, Additional file 2: Figure S2).

**Table 1:** Characteristics and outcomes of the overall cohort of patients treated with noninvasive ventilation for de novo acute hypoxemic respiratory failure

	Not immunocompromised (*n* = 137)	Immunocompromised (*n* = 71)	*p* value
***Demographic characteristics***			
Age, years	64 (51–76)	56 (47–67)	0.08
Gender, male, *n* (%)	88 (64%)	50 (70%)	0.46
Simplified acute physiology score II	34 (27–42)	40 (33–49)	0.001
***Risk factor for acute respiratory failure, n (%)***			0.46
Pulmonary	103 (75%)	59 (83%)	
No risk factor	19 (14%)	9 (13%)	
Extrapulmonary	15 (11%)	3 (4.2%)	
***Bilateral lung infiltrates, n (%)***	103 (75%)	67 (94%)	0.001
***Under oxygen***			
Glasgow score	15 (15–15)	15 (15–15)	0.19
Systolic blood pressure, mmHg	128 (110–144)	130 (116–141)	0.97
Heart rate, per min	105 (92–120)	110 (99–124)	0.15
Respiratory rate, per min	31 (27–36)	32 (28–37)	0.33
Oxygen flow, L/min	15 (10–15)	12 (6–15)	0.29
PaO_2_/FiO_2_, mmHg	122 (89–176)	135 (96–194)	0.54
PaCO_2_, mmHg	36 (31–39)	34 (31–37)	0.28
pH	7.44 (7.41–7.47)	7.46 (7.42–7.48)	0.43
***Under noninvasive ventilation after 1 h***			
Pressure support, cm H_2_O	8 (6–9)	8 (7–10)	0.02
Positive end-expiratory pressure, cm H_2_O	5 (5–5)	5 (5–5)	0.56
FiO_2_, %	70 (50–100)	80 (53–100)	0.056
SpO_2_, %	97 (95–99)	98 (96–100)	0.052
Respiratory rate, per min	30 (24–35)	30 (24–39)	0.19
Expired tidal volume, mL	577 (471–662)	622 (526–764)	0.07
Minute ventilation, L/min	16.3 (13.2–20.2)	19.2 (15.3–24.0)	0.02
PaO_2_/FiO_2_, mmHg	164 (114–230)	181 (121–261)	0.24
< 150 mmHg, *n* (%)	52/121 (43%)	27/70 (39%)	0.66
PaCO_2_, mmHg	36 (32–40)	35 (30–40)	0.33
pH	7.43 (7.39–7.47)	7.45 (7.40–7.49)	0.56
***Under noninvasive ventilation within the first 24 h after ICU admission***			
Worst PaO_2_/FiO_2_, mmHg	130 (98–182)	130 (91–181)	0.91
< 150 mmHg, *n* (%)	71/123 (58%)	46/70 (66%)	0.35
Acute respiratory distress syndrome, *n* (%)	99 (72%)	63 (89%)	0.01
***Outcomes***			
Intubation, *n* (%)	59 (43%)	43 (61%)	0.02
Time to intubation, h	5 (2–21)	6 (2–48)	0.22
ICU mortality, *n* (%)	20 (15%)	27 (38%)	0.0003
ICU mortality of intubated patients	20/59 (37%)	27/43 (63%)	0.007
ICU length of stay, day	9 (6–16)	9 (6–17)	0.96

### Factors associated with NIV failure and in-ICU mortality in the overall population

Using univariate analysis, factors associated with NIV failure and in-ICU mortality were similar and included SAPS II, immunosuppression, bilateral lung infiltrates, ARDS, and the following variables 1 h after NIV initiation: high FiO_2_, large tidal volumes, high minute ventilation, and low PaO_2_/FiO_2_ (Additional file 3: Table S1, Additional file 4: Table S2). After adjustment, expired tidal volume and PaO_2_/FiO_2_ 1 h after NIV initiation were associated with NIV failure, whereas immunosuppression status was not anymore (Table 2). Conversely, factors independently associated with mortality included expired tidal volume 1 h after NIV initiation and immunosuppression status (Table 2).

**Table 2:** Multivariate analysis of variables associated with intubation and mortality in the overall population and in the propensity score-matched cohort

Variables	Adjusted odds ratio (95% confidence interval)	*p* value
***In the overall population***		
*Factors associated with intubation* ^a^		
Expired tidal volume after 1 h under noninvasive ventilation, per 100 mL increase	1.27 (1.03–1.58)	0.03
PaO_2_/FiO_2_ after 1 h under noninvasive ventilation, per mmHg drop	1.006 (1.002–1.010)	0.003
*Factors associated with ICU mortality* ^b^		
Immunodepression	2.64 (1.24–5.67)	0.01
Expired tidal volume after 1 h of noninvasive ventilation, per 100 mL increase	1.37 (1.09–1.74)	0.008
***In the propensity score-matched cohort***		
*Factors associated with intubation* ^c^		
Heart rate, per beat per min drop	1.02 (1.001–1.01)	0.048
PaO_2_/FiO_2_ after 1 h of noninvasive ventilation, per mmHg drop	1.007 (1.002–1.011)	0.007
*Factors associated with ICU mortality* ^d^		
Immunosuppression	2.56 (1.54–6.57)	0.04
Heart rate, per beat per min drop	1.03 (1.01–1.05)	0.01

^a^Variables included in the backward stepwise logistic regression model were age, immunosuppression, Glasgow score of oxygen, bilateral lung infiltrates, expired tidal volume after 1 h under noninvasive ventilation, PaO_2_/FiO_2_ after 1 h under noninvasive ventilation

^b^Variables included in the backward stepwise logistic regression model were age, immunosuppression, bilateral lung infiltrates, expired tidal volume after 1 h of noninvasive ventilation, PaO_2_/FiO_2_ after 1 h of noninvasive ventilation

^c^Variables included in the backward stepwise logistic regression model were age, systolic blood pressure under oxygen, heart rate under oxygen, and PaO_2_/FiO_2_ after 1 h under noninvasive ventilation. Immunosuppression was forced into the model

^d^Variables included in the backward stepwise logistic regression model were age, immunosuppression, heart rate under oxygen, tidal volume under noninvasive ventilation, PaO_2_/FiO_2_ after 1 h of noninvasive ventilation

### Propensity score-matched cohort analysis

**Fig. 2:**
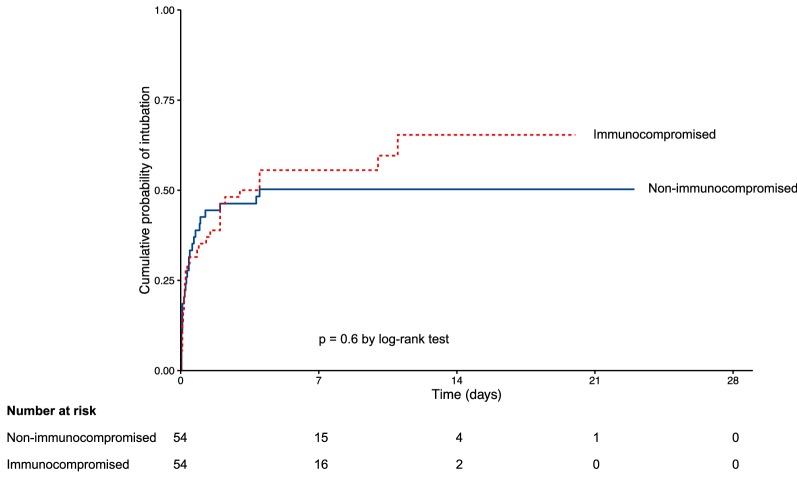
Kaplan-Meier curves of the cumulative probability of intubation in the propensity score matched cohort. Curves were compared using the log-rank test

**Fig. 3:**
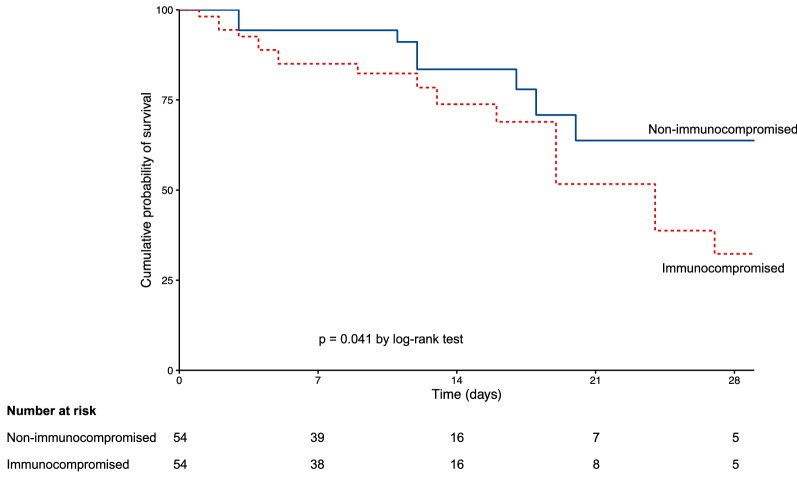
Kaplan-Meier curves of the cumulative probability of survival in the propensity score matched cohort. Curves were compared using the log-rank test

The propensity score was computed based on age, gender, bilateral lung infiltrates, and the following variables 1 h after NIV initiation: pressure-support level, tidal volume, minute ventilation, FiO_2_, and PaO_2_/FiO_2_ < 150 mmHg. In the matched cohort, PaO_2_/FiO_2_ under oxygen was higher in immunocompromised than in nonimmunocompromised patients and there was no other difference between groups (Additional file 5: Table S3). Variables associated with intubation and mortality in the matched cohort are displayed in Additional file 6: Table S4 and Additional file 7: Table S5. Using log-rank test in the matched cohort, cumulative intubation rate was not different between immunocompromised and immunocompetent patients using log-rank test (*p* = 0.6, Fig. 2). Conversely, cumulative probability for survival remained higher in immunocompromised than in immunocompromised patients (*p* = 0.04, Fig. 3). After adjustment, heart rate under oxygen and PaO_2_/FiO_2_ 1 h after NIV initiation (Table 2) were independently associated with intubation. Immunosuppression was not independently associated with NIV failure even after being forced into the model, whereas it remained independently associated with mortality (Table 2).

## Discussion

The direct influence of immunosuppression status on outcomes of patients with acute hypoxemic respiratory failure treated with NIV remains poorly known. Our main results are that immunosuppression status was strongly associated with mortality but not with intubation after adjustment, nor after matching on markers of respiratory disease severity. Indeed, PaO_2_/FiO_2_ and large tidal volumes 1 h after NIV initiation were stronger predictors of NIV failure than immunosuppression status per se. By contrast, once intubated after NIV failure, mortality was markedly higher in immunocompromised patients than in the others.

In our cohort, immunocompromised patients treated with NIV required intubation in 61% of cases, knowing that the vast majority (89%) met the criteria for ARDS [26]. Our findings are in keeping with these from Azoulay and colleagues, who reported NIV failure rates of 71% in a pooled analysis including 387 immunocompromised patients with ARDS [28], and with the 63% failure rate reported in a sub-analysis of a large international cohort study on ARDS [21]. Similarly, mortality rates in these two cohorts were 44 and 43% [21, 29], i.e., very close to the 38% we report, thereby reinforcing the external validity of our results.

Immunosuppression status was associated with a 2.6-fold risk for mortality after adjustment. It has been extensively reported to be an independent risk factor for mortality in patients treated with NIV for ARDS or influenza [30–32]. By contrast, whether immunosuppression is a risk for NIV failure has been poorly studied. Despite higher intubation rates in immunocompromised than in nonimmunocompromised patients, immunosuppression status did not remain associated with intubation after adjustment on variables of respiratory disease severity such as hypoxemia and large tidal volumes generated under NIV. These results are in line with previous studies reporting that immunosuppression was associated with NIV failure by univariate analysis, but not after adjustment [33, 34]. However, in the first study, adjustment was performed on SAPS II, which shares collinearity with immunosuppression [33]. Moreover, in the second study, most of the patients had cardiogenic pulmonary edema or acute-on-chronic respiratory failure [34], in whom strong benefits of NIV have been reported [35, 36]. Due to the above-mentioned limitations, the impact of immunosuppression status on NIV failure rate could have been mitigated. However, the absence of association between immunosuppression status and NIV failure after adjustment in both our overall cohort and in our propensity score-matched cohort suggests that immunosuppression status is not per se a risk factor for NIV failure, and that immunocompromised patients might have been more severe than nonimmunocompromised at inclusion despite similar vital signs, oxygen requirements, and arterial blood gas at baseline. Whether similar results would have been observed with less severe patients (such as patients with less oxygen requirement or less hypoxemia) is unclear. Indeed, although oxygen requirement at ICU admission is independently associated with mortality in immunocompromised patients, its effect on intubation has not been tested yet [37].

After adjustment, mortality rate was higher in immunocompromised than in nonimmunocompromised patients despite similar NIV failure rates. As patients with a do-not-intubate order were excluded from the analysis, this striking difference pertains exclusively to different mortality rates of intubated patients. This difference could be related to differences in respiratory mechanics under invasive ventilation between the two groups, such as driving pressure, an important determinant of ARDS prognosis [38]. Unfortunately, driving pressure of the respiratory system was not collected in the two studies. Likewise, the cause of death was not collected and it is possible that the proportion of patients with limitation of life-sustaining measures was different between the two groups [21]. Last, we cannot rule out some differences in the management of invasive ventilation between the two groups given it was not protocolized in the two studies. However, we believe that patients studied were managed according to the best practice given that all participating centers are experienced in the field of invasive ventilation, and have been involved in several trials on invasive ventilation. Our findings highlight the urge for standardization of invasive ventilation settings, collection of respiratory mechanics, and the cause of death, even in studies comparing two first-line, early, noninvasive ventilation strategies to better understand the influence of one strategy over the other (NCT02978300).

PaO_2_/FiO_2_ 1 h after NIV initiation was independently associated with NIV failure. Hypoxemia is a well-known risk factor for NIV failure. In line with our findings, two prospective cohort studies have reported that PaO_2_/FiO_2_ lower than 146 mmHg and 175 mmHg after 1 h of NIV was independently associated with NIV failure [39, 40]. Likewise, the HACOR score proposed by Duan and colleagues to predict NIV failure allocated a stepwise increasing score for PaO_2_/FiO_2_ drop 1 h after NIV initiation [41]. A similar association between hypoxemia and NIV failure had previously been reported [30, 31]. However, the timing of hypoxemia assessment was unclear, and we believe that early evaluation (1 h after NIV initiation, for instance) is important to avoid the harmful effects of late intubation [42]. All in all, PaO_2_/FiO_2_ 1 h after NIV initiation seems an interesting early predictor of NIV failure and mortality. As a consequence, patients remaining hypoxemic after 1 h of NIV should be monitored closely to avoid the deleterious effects of spontaneous breathing [43].

Large tidal volumes 1 h after NIV initiation were also independently associated with NIV failure. Although tidal volumes were not normalized to the patient’s predicted body weight due to missing data, our results are in line with a recent study reporting that large expired tidal volumes were independent predictors of NIV failure in de novo acute hypoxemic respiratory failure [33]. Interestingly, large tidal volumes 1 h after NIV initiation were also associated with mortality. This finding may illustrate the concept of patient self-inflicted lung injury [43]. Spontaneously breathing patients with acute respiratory failure have a high respiratory drive, which generates large transpulmonary driving pressures and subsequent large tidal volumes, which stretch the lungs and may worsen lung injury [44]. Our results suggest that large tidal volumes and low PaO_2_/FiO_2_ are more important risk factors for NIV failure than immunosuppression status by itself, and that particular attention should be paid to large tidal volumes and blood gas analysis as early as 1 h after NIV initiation in patients with de novo acute hypoxemic respiratory failure.

Some limitations have to be acknowledged. First, this is an unplanned post hoc analysis of two studies with inherent possible bias. Although in the study from Thille and colleagues, clinical data were collected retrospectively based on computerized medical charts, NIV settings were collected prospectively, thereby reducing potential selection and information bias. Moreover, NIV failure and mortality rates are in line with previous studies suggesting that our results might be generalizable. Second, the sample size is relatively small and our results deserve confirmation in larger multicenter prospective cohorts. Third, it is worth noting that patients from the Frat study received high-flow oxygen therapy, whereas those from the Thille study received standard oxygen therapy during NIV breaks [11, 22]. This difference was not considered in our analysis because we did not collect data between NIV sessions (data were exclusively collected under standard oxygen therapy before ICU admission or under NIV after ICU admission). Moreover, in a previous study on immunocompromised patients, intubation and mortality rates of patients treated with high-flow or standard oxygen therapy during NIV breaks were not different [45]. Fourth, the choice of variables included in multivariate analysis could be argued. We chose early easy-to-assess variables, rather than variables to be calculated 24 h after ICU admission such as SAPS II [24]. Indeed, given the harm of delayed NIV failure [42], it seems important to identify as early as possible the factors associated with poor outcomes. Fifth, although our results suggest that immunosuppression status is independently associated with mortality in patients with de novo acute hypoxemic respiratory failure treated with NIV, whether a different protocol to carry out NIV aiming at more protective ventilation (high PEEP, low pressure support) would lead to different results is unknown. Indeed, a recent randomized trial including patients with ARDS, half of whom were immunocompromised, reported better outcomes when NIV was applied with a helmet and high PEEP levels than with a face mask and lower PEEP levels [46].

## Conclusion

This post hoc analysis of two studies including 34% of immunocompromised patients with de novo acute hypoxemic respiratory failure treated with NIV suggests that immunosuppression was independently associated with mortality but not with intubation after adjustment. Large tidal volumes and hypoxemia 1 h after NIV initiation seemed to be stronger predictors of NIV failure than immunosuppression status. These results deserve to be confirmed in a larger sample size prospective multicenter study.

## Additional files

**Additional file 1: Figure S1.** Kaplan–Meier curves of the cumulative probability of intubation in the overall population.

**Additional file 2: Figure S2.** Kaplan–Meier curves of the cumulative probability of survival in the overall population.

**Additional file 3: Table S1.** Univariate analysis of variables associated with intubation in the overall population of patients treated with noninvasive ventilation for *de novo* acute hypoxemic respiratory failure.

**Additional file 4: Table S2.** Univariate analysis of variables associated with mortality in the overall population of patients treated with noninvasive ventilation for *de novo* acute hypoxemic respiratory failure.

**Additional file 5: Table S3.** Characteristics and outcomes of the 108 patients treated with noninvasive ventilation for acute hypoxemic respiratory failure in the propensity score matched cohort.

**Additional file 6: Table S4.** Univariate analysis of variables associated with intubation in the propensity score matched cohort.

**Additional file 7: Table S5.** Univariate analysis of variables associated with mortality in the propensity score matched cohort.

## Data Availability

All data analyzed during this study are included in this published article [and its additional information files].

## Abbreviations

ARDS: acute respiratory distress syndrome
FiO_2_: fraction of inspired oxygen
ICU: intensive care unit
NIV: noninvasive ventilation
PaCO_2_: partial pressure of carbon dioxide in arterial blood
PaO_2_: partial pressure of oxygen in arterial blood
PEEP: positive end-expiratory pressure
SAPS II: simplified acute physiology score II
SD: standard deviation
Vte: expired tidal volume
