# Supplementary figures and images for: Is immunosuppression status a risk factor for noninvasive ventilation failure in patients with acute hypoxemic respiratory failure? A post hoc matched analysis
Source: Ann Intensive Care. 2019 Aug 14;9:90. doi: 10.1186/s13613-019-0566-z (PMC6692798; doi:10.1186/s13613-019-0566-z)

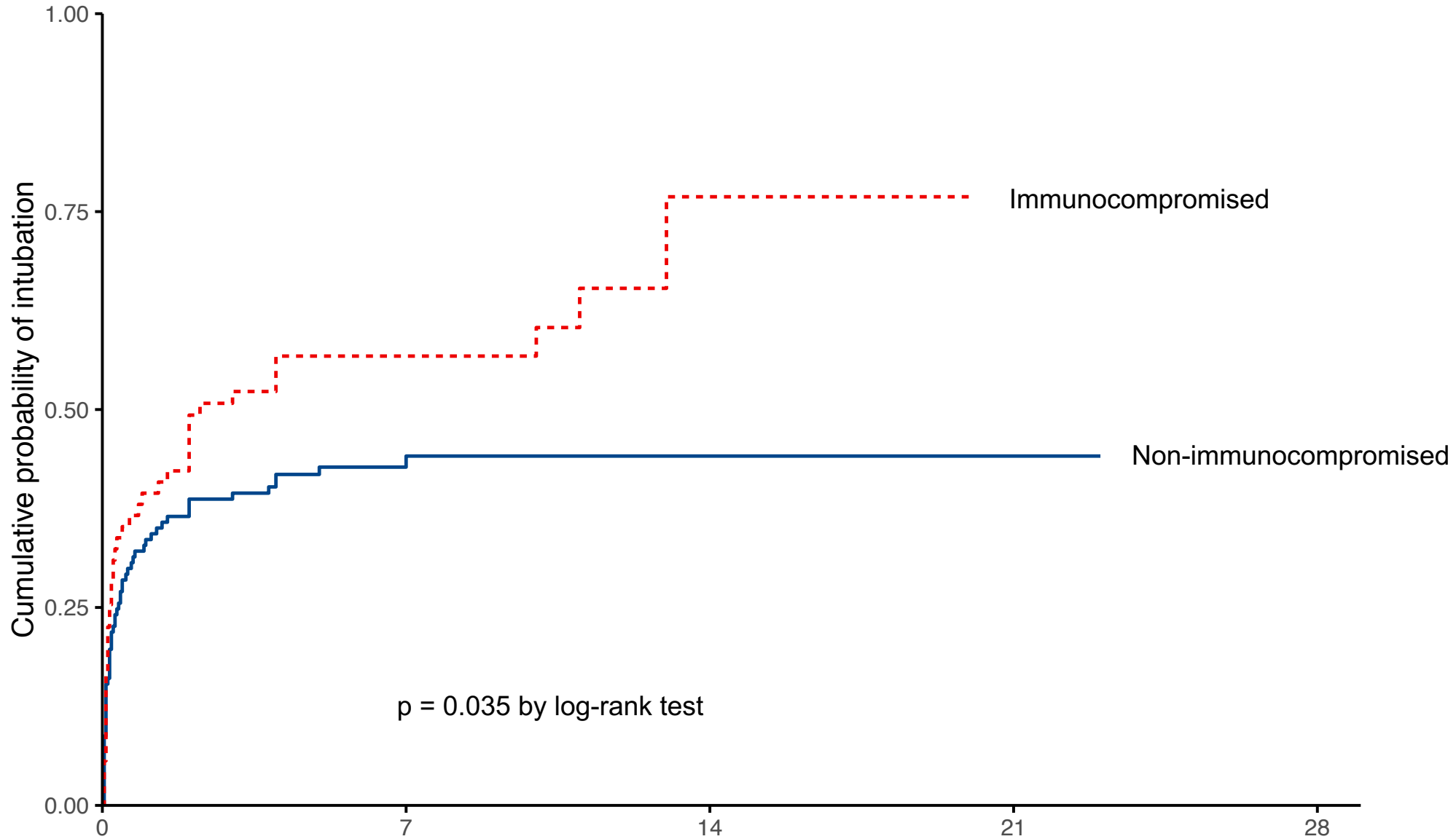

**Number at risk**

|                       |     |    |    |   |   |
|-----------------------|-----|----|----|---|---|
| Non-immunocompromised | 137 | 41 | 11 | 1 | 0 |
| Immunocompromised     | 71  | 18 | 2  | 0 | 0 |

Supplement: Supplementary file 1 — Additional file 1: Figure S1. Kaplan–Meier curves of the cumulative probability of intubation in the overall population. [file 13613_2019_566_MOESM1_ESM.pdf]

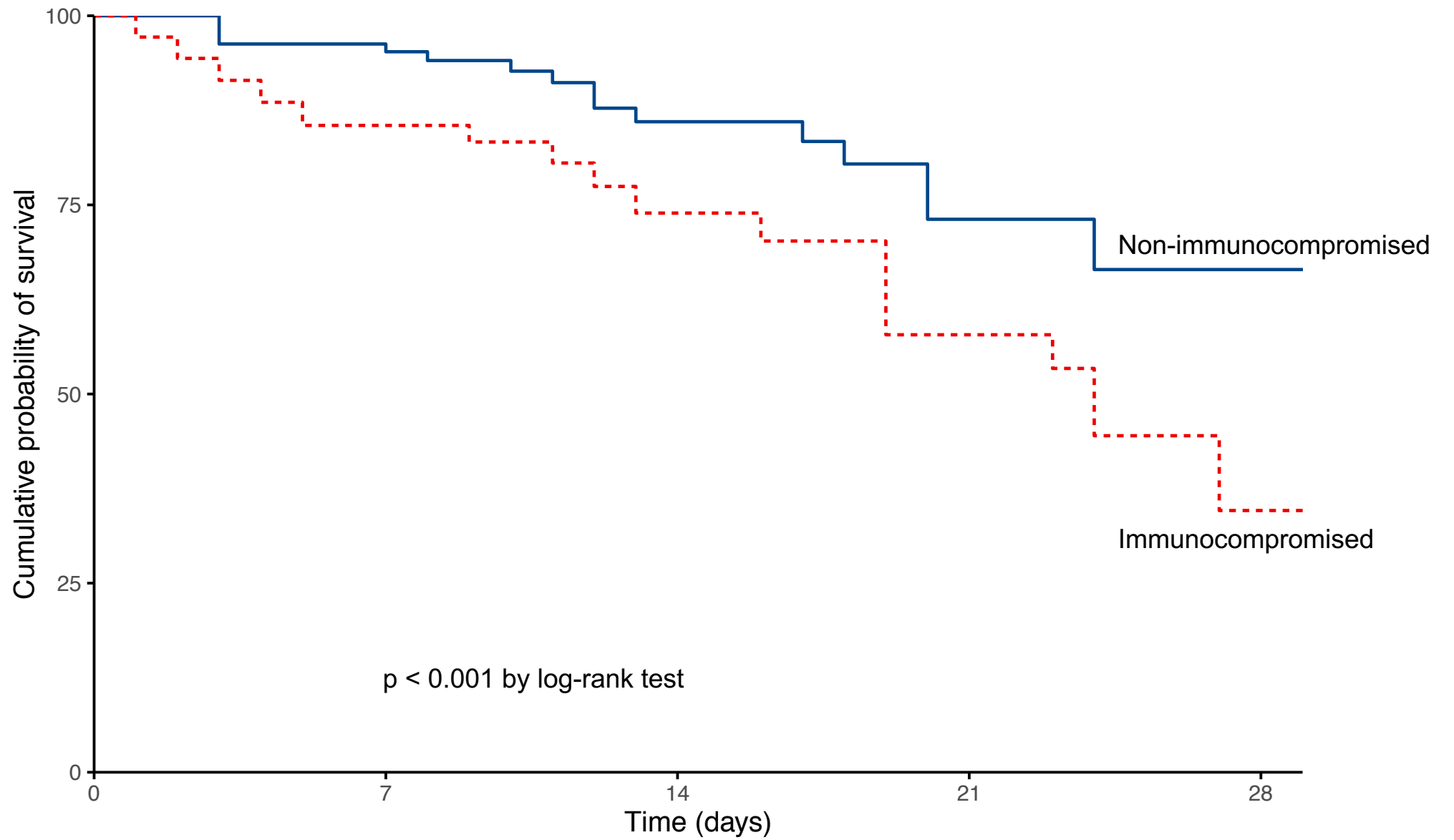

**Number at risk**

|                       |     |    |    |    |   |
|-----------------------|-----|----|----|----|---|
| Non-immunocompromised | 137 | 94 | 43 | 17 | 9 |
| Immunocompromised     | 71  | 48 | 21 | 13 | 7 |

Supplement: Supplementary file 2 — Additional file 2: Figure S2. Kaplan–Meier curves of the cumulative probability of survival in the overall population. [file 13613_2019_566_MOESM2_ESM.pdf]
