# Supplementary material for: Is immunosuppression status a risk factor for noninvasive ventilation failure in patients with acute hypoxemic respiratory failure? A post hoc matched analysis
Source: Ann Intensive Care. 2019 Aug 14;9:90. doi: 10.1186/s13613-019-0566-z (PMC6692798; doi:10.1186/s13613-019-0566-z)
Supplement: Supplementary file 3 — Additional file 3: Table S1. Univariate analysis of variables associated with intubation in the overall population of patients treated with noninvasive ventilation for de novo acute hypoxemic respiratory failure. [file 13613_2019_566_MOESM3_ESM.docx]

**Additional Table S1. Univariate analysis of variables associated with intubation in the overall population of patients treated with noninvasive ventilation for *de novo* acute hypoxemic respiratory failure.**

|  | **Not intubated**  **(n=106)** | **Intubated**  **(n=102)** | **P value** |
| --- | --- | --- | --- |
| Demographic characteristics | | | |
| Age, years | 59 (48-74) | 63 (51-73) | 0.12 |
| Gender, male, n (%) | 72 (68%) | 66 (65%) | 0.73 |
| Simplified acute physiology score 2 | 31 (23-39) | 41 (33-48) | <0.0001 |
| Immunocompromised, n (%) | 28 (26%) | 43 (42%) | 0.02 |
| Risk factor for acute respiratory failure, n (%) | | | 0.15 |
| Pulmonary | 78 (74%) | 84 (82%) | 0.18 |
| Extrapulmonary | 15 (14%) | 5 (4.9%) | 0.11 |
| No risk factor | 13 (12%) | 13 (13%) | 0.93 |
| Bilateral lung infiltrates, n (%) | 80 (75%) | 90 (88%) | 0.03 |
| Under oxygen | | | |
| Glasgow score | 15 (15-15) | 15 (15-15) | 0.15 |
| Systolic blood pressure, mm Hg | 129 (110-148) | 127 (115-141) | 0.34 |
| Heart rate, per min | 109 (93-121) | 107 (94-120) | 0.83 |
| Respiratory rate, per min | 31 (28-35) | 33 (28-38) | 0.43 |
| Oxygen flow, l/min | 15 (8-15) | 13 (10-15) | 0.43 |
| PaO_2_/FiO_2_, mm Hg | 133 (95-195) | 120 (86-179) | 0.66 |
| PaCO_2_, mm Hg | 35 (32-39) | 35 (30-39) | 0.88 |
| pH | 7.44 (7.39-7.47) | 7.46 (7.42-7.49) | 0.16 |
| Under noninvasive ventilation after 1 hour | | | |
| Pressure support, cm H_2_O | 8 (7-10) | 8 (6-10) | 0.97 |
| Positive end-expiratory pressure, cm H_2_O | 5 (5-5) | 5 (5-5) | 0.12 |
| FiO_2_, % | 60 (50-100) | 90 (56-100) | 0.01 |
| SpO_2_, % | 98 (96-100) | 97 (95-99) | 0.14 |
| Respiratory rate, per min | 28 (24-35) | 32 (26-37) | 0.10 |
| Expired tidal volume, mL | 546 (455-653) | 624 (516-742) | 0.008 |
| Minute ventilation, L/min | 15.5 (11.8-20.0) | 19.1 (15.7-23.3) | 0.007 |
| PaO_2_/FiO_2_, mm Hg | 202 (145-266) | 146 (100-198) | 0.009 |
| < 150 mm Hg, n (%) | 27/94 (29%) | 52/97 (54%) | 0.0001 |
| PaCO_2_, mm Hg | 35 (32-39) | 35 (30-39) | 0.55 |
| pH | 7.44 (7.40-7.47) | 7.44 (7.40-7.48) | 0.99 |
| Under noninvasive ventilation within the first 24 hours after ICU admission | | | |
| Worst PaO_2_/FiO_2_, mm Hg | 164 (118-223) | 104 (81-145) | <0.0001 |
| < 150 mm Hg, n (%) | 42/96 (44%) | 75/97 (77%) | 0.0008 |
| Acute respiratory distress syndrome, n (%) | 73 (69%) | 89 (87%) | 0.002 |
| Outcomes |  |  |  |
| ICU mortality, n (%) | 0 (0.0%) | 47 (46%) | <0.0001 |
| ICU length of stay, d | 7 (5-10) | 14 (8-23) | <0.0001 |
