# Supplementary material for: Is immunosuppression status a risk factor for noninvasive ventilation failure in patients with acute hypoxemic respiratory failure? A post hoc matched analysis
Source: Ann Intensive Care. 2019 Aug 14;9:90. doi: 10.1186/s13613-019-0566-z (PMC6692798; doi:10.1186/s13613-019-0566-z)
Supplement: Supplementary file 4 — Additional file 4: Table S2. Univariate analysis of variables associated with mortality in the overall population of patients treated with noninvasive ventilation for de novo acute hypoxemic respiratory failure. [file 13613_2019_566_MOESM4_ESM.docx]

**Additional Table S2. Univariate analysis of variables associated with mortality in the overall population of patients treated with noninvasive ventilation for *de novo* acute hypoxemic respiratory failure.**

|  | **Survivors**  **(n=161)** | **Non-survivors**  **(n=47)** | **P value** |
| --- | --- | --- | --- |
| Demographic characteristics | | | |
| Age, years | 60 (49-74) | 66 (56-72) | 0.10 |
| Gender, male, n (%) | 104 (65%) | 34 (72%) | 0.42 |
| Simplified acute physiology score 2 | 33 (26-41) | 42 (36-50) | <0.0001 |
| Immunocompromised, n (%) | 44 (27%) | 27 (57%) | 0.0003 |
| Risk factor for acute respiratory failure, n (%) | | | 0.45 |
| Pulmonary | 123 (76%) | 39 (83%) |  |
| Extrapulmonary | 16 (9.9%) | 2 (4.3%) |  |
| No risk factor | 22 (14%) | 6 (13%) |  |
| Bilateral lung infiltrates, n (%) | 126 (78%) | 44 (94%) | 0.03 |
| Under oxygen | | | |
| Glasgow score | 15 (15-15) | 15 (15-15) | 0.61 |
| Systolic blood pressure, mm Hg | 130 (112-147) | 124 (116-139) | 0.24 |
| Heart rate, per min | 109 (96-123) | 105 (92-118) | 0.27 |
| Respiratory rate, per min | 31 (28-36) | 34 (28-38) | 0.37 |
| Oxygen flow, l/min | 12 (9-15) | 15 (10-15) | 0.42 |
| PaO_2_/FiO_2_, mm Hg | 131 (95-180) | 116 (78-185) | 0.71 |
| PaCO_2_, mm Hg | 35 (31-39) | 34 (32-38) | 0.91 |
| pH | 7.45 (7.41-7.47) | 7.46 (7.42-7.49) | 0.22 |
| Under noninvasive ventilation after 1 hour | | | |
| Pressure support, cm H_2_O | 8 (6-10) | 8 (7-10) | 0.80 |
| Positive end-expiratory pressure, cm H_2_O | 5 (5-5) | 5 (5-5) | 0.12 |
| FiO_2_, % | 70 (50-100) | 100 (60-100) | 0.01 |
| SpO_2_, % | 98 (96-99) | 98 (95-99) | 0.56 |
| Respiratory rate, per min | 29 (24-35) | 32 (26-37) | 0.26 |
| Expired tidal volume, mL | 560 (470-652) | 660 (579-751) | 0.003 |
| Minute ventilation, L/min | 16.3 (13.0-20.3) | 20.6 (16.4-24.0) | 0.003 |
| PaO_2_/FiO_2_, mm Hg | 179 (121-242) | 142 (106-203) | 0.07 |
| < 150 mm Hg, n (%) | 53/144 (37%) | 26 (55%) | 0.04 |
| PaCO_2_, mm Hg | 36 (32-40)6 | 36 (30-42) | 0.69 |
| pH | 7.44 (7.39-7.47) | 7.45 (7.40-7.48) | 0.44 |
| Under noninvasive ventilation within the first 24 hours after ICU admission | | | |
| Worst PaO_2_/FiO_2_, mm Hg | 140 (102-200) | 104 (82-133) | <0.0001 |
| Worst PaO_2_/FiO_2_ < 150 mm Hg, n (%) | 80/146 (55%) | 37 (79%) | 0.006 |
| Acute respiratory distress syndrome, n (%) | 118 (73%) | 44 (94%) | 0.006 |
| Outcomes |  |  |  |
| Intubation, n (%) | 55 (34%) | 47 (100%) | <0.0001 |
| Time to intubation (h) | 5 (2-28) | 7 (2-31) | 0.60 |
| ICU length of stay, d | 8 (6-14) | 12 (4-24) | 0.15 |
