# Supplementary material for: Is immunosuppression status a risk factor for noninvasive ventilation failure in patients with acute hypoxemic respiratory failure? A post hoc matched analysis
Source: Ann Intensive Care. 2019 Aug 14;9:90. doi: 10.1186/s13613-019-0566-z (PMC6692798; doi:10.1186/s13613-019-0566-z)
Supplement: Supplementary file 5 — Additional file 5: Table S3. Characteristics and outcomes of the 108 patients treated with noninvasive ventilation for acute hypoxemic respiratory failure in the propensity score matched cohort. [file 13613_2019_566_MOESM5_ESM.docx]

**Additional Table S3. Characteristics and outcomes of the 108 patients treated with noninvasive ventilation for acute hypoxemic respiratory failure in the propensity score matched cohort.**

|  | **Not Immunocompromised**  **(n=54)** | **Immunocompromised**  **(n=54)** | **P value** |  |
| --- | --- | --- | --- | --- |
| Demographic characteristics | | | | |
| Age, years | 60 (45-74) | 57 (47-67) | 0.93 |  |
| Gender, male, n (%) | 38 (70%) | 40 (74%) | 0.82 |  |
| Simplified acute physiology score II | 34 (25-43) | 38 (32-48) | 0.06 |  |
| Risk factor for acute respiratory failure, n (%) |  |  | 0.44 |  |
| Pulmonary | 39 (72%) | 44 (81%) |  |  |
| No risk factor | 7 (13%) | 7 (13%) |  |  |
| Extrapulmonary | 8 (15%) | 3 (5.6%) |  |  |
| Bilateral lung infiltrates, n (%) | 53 (98%) | 53 (98%) | >0.99 |  |
| Under oxygen | | | | |
| Glasgow score | 15 (15-15) | 15 (15-15) | 0.78 |  |
| Systolic blood pressure, mm Hg | 132 (119-150) | 130 (117-140) | 0.15 |  |
| Heart rate, per min | 109 (96-125) | 110 (96-123) | 0.51 |  |
| Respiratory rate, per min | 30 (27-38) | 32 (28-37) | 0.79 |  |
| Oxygen flow, l/min | 14 (10-15) | 12 (6-15) | 0.10 |  |
| PaO_2_/FiO_2_, mm Hg | 109 (86-152) | 148 (100-194) | 0.04 |  |
| PaCO_2_, mm Hg | 36 (31-39) | 34 ± 5 | 0.85 |  |
| pH | 7.44 (7.40-7.47) | 7.44 ± 0.07 | 0.60 |  |
| Under noninvasive ventilation after 1 hour | | | | |
| Respiratory rate, per min | 31 (24-37) | 32 (28-37) | 0.48 |  |
| SpO_2_, % | 98 (96-99) | 98 (96-100) | 0.42 |  |
| Expired tidal volume, mL | 599 (503-729) | 622 (523-765) | 0.68 |  |
| Minute ventilation, L/min | 18.6 (15.0-21.3) | 19.1 (15.3-24.1) | 0.34 |  |
| Pressure support, cm H_2_O | 8 (7-8) | 8 (7-10) | 0.06 |  |
| Positive end-expiratory pressure, cm H_2_O | 5 (5-5) | 5 (5-5) | 0.28 |  |
| FiO_2_, % | 80 (50-100) | 80 (50-100) | 0.65 |  |
| PaO_2_/FiO_2_, mm Hg | 174 (123-259) | 186 (123-266) | 0.81 |  |
| PaO_2_/FiO_2_ < 150 mm Hg, n (%) | 17 (31%) | 18 (33%) | >0.99 |  |
| PaCO_2_, mm Hg | 37 (31-40) | 36 (31-41) | 0.87 |  |
| pH | 7.42 (7.38-7.47) | 7.46 (7.42-7.48) | 0.93 |  |
| Under noninvasive ventilation within the first 24 hours after ICU admission | | | | |
| Worst PaO_2_/FiO_2_, mm Hg | 150 (105-214) | 130 (88-174) | 0.34 |  |
| Worst PaO_2_/FiO_2_ < 150 mm Hg, n (%) | 26 (48%) | 18 (33%) | 0.11 |  |
| Acute respiratory distress syndrome, n (%) | 51 (94%) | 50 (93%) | >0.99 |  |
| Outcomes | | | | |
| Intubation, n (%) | 27 (50%) | 32 (59%) | 0.50 |  |
| Time to intubation, h | 7 (2-13) | 10 (3-48) | 0.37 |  |
| ICU mortality, n (%) | 10 (19%) | 20 (37%) | 0.09 |  |
| ICU length of stay, d | 11 (6-16) | 10 (6-16) | 0.78 |  |
