# Supplementary material for: Is immunosuppression status a risk factor for noninvasive ventilation failure in patients with acute hypoxemic respiratory failure? A post hoc matched analysis
Source: Ann Intensive Care. 2019 Aug 14;9:90. doi: 10.1186/s13613-019-0566-z (PMC6692798; doi:10.1186/s13613-019-0566-z)
Supplement: Supplementary file 6 — Additional file 6: Table S4. Univariate analysis of variables associated with intubation in the propensity score matched cohort. [file 13613_2019_566_MOESM6_ESM.docx]

**Additional Table S4. Univariate analysis of variables associated with intubation in the propensity score matched cohort.**

|  | **Not intubated**  **(n=49)** | **Intubated**  **(n=59)** | | **P value** | |
| --- | --- | --- | --- | --- | --- |
| Demographic characteristics | | | | |  |
| Age, years | 54 (40-68) | 61 (51-71) | | 0.11 | |
| Gender, male, n (%) | 36 (73%) | 42 (71%) | | 0.96 | |
| Simplified acute physiology score 2 | 31 (23-38) | 41 (32-49) | | <0.001 | |
| Immunocompromised, n (%) | 22 (45%) | 32 (54%) | | 0.44 | |
| Risk factor for acute respiratory failure, n (%) | | | 0.36 | |  |
| Pulmonary | 37 (76%) | 46 (78%) | |  | |
| Extrapulmonary | 7 (14%) | 4 (6.8%) | |  | |
| No risk factor | 5 (10%) | 9 (15%) | |  | |
| Bilateral lung infiltrates, n (%) | 48 (98%) | 58 (98%) | | >0.99 | |
| Under oxygen | | | | |  |
| Glasgow score | 15 (15-15) | 15 (15-15) | | 0.42 | |
| Systolic blood pressure, mm Hg | 132 (119-154) | 131 (116-140) | | 0.09 | |
| Heart rate, per min | 114 (99-125) | 105 (94-121) | | 0.06 | |
| Respiratory rate, per min | 32 (29-37) | 30 (27-38) | | 0.51 | |
| Oxygen flow, l/min | 12 (6-15) | 12 (10-15) | | 0.32 | |
| PaO_2_/FiO_2_, mm Hg | 133 (96-194) | 115 (92-159) | | 0.26 | |
| PaCO_2_, mm Hg | 35 (32-37) | 34 (30-38) | | 0.93 | |
| pH | 7.44 (7.40-7.47) | 7.46 (7.42-7.48) | | 0.49 | |
| Under noninvasive ventilation after 1 hour | | | | |  |
| Respiratory rate, per min | 28 (24-38) | 32 (24-38) | | 0.72 | |
| SpO_2_, % | 98 (96-100) | 97 (96-99) | | 0.10 | |
| Expired tidal volume, mL | 580 (491-735) | 630 (539-759) | | 0.19 | |
| Minute ventilation, L/min | 16.3 (14.6-21.7) | 20.1 (16.5-23.8) | | 0.18 | |
| Pressure support, cm H_2_O | 8 (7-9) | 8 (7-10) | | 0.91 | |
| Positive end-expiratory pressure, cm H_2_O | 5 (5-6) | 5 (4-5) | | 0.005 | |
| FiO_2_, % | 75 (50-100) | 100 (58-100) | | 0.20 | |
| PaO_2_/FiO_2_, mm Hg | 225 (167-274) | 153 (112-234) | | 0.006 | |
| PaO_2_/FiO_2_ < 150 mm Hg, n (%) | 8 (16%) | 27 (46%) | | 0.002 | |
| PaCO_2_, mm Hg | 38 (33-40) | 36 (30-42) | | 0.94 | |
| pH | 7.44 (7.40-7.46) | 7.44 (7.39-7.47) | | 0.95 | |
| Under noninvasive ventilation within the first 24 hours after ICU admission | | | | |  |
| Worst PaO_2_/FiO_2_, mm Hg | 172 (140-232) | 117 (183-144) | | <0.001 | |
| Worst PaO_2_/FiO_2_ < 150 mm Hg, n (%) | 16 (33%) | 46 (78%) | | <0.001 | |
| Acute respiratory distress syndrome, n (%) | 44 (90%) | 57 (97%) | | 0.30 | |
| Outcomes |  |  | |  | |
| ICU mortality, n (%) | 0 (0%) | 30 (51%) | | <0.001 | |
| ICU length of stay, d | 7 (6-11) | 13 (8-20) | | <0.001 | |
