# Supplementary material for: Is immunosuppression status a risk factor for noninvasive ventilation failure in patients with acute hypoxemic respiratory failure? A post hoc matched analysis
Source: Ann Intensive Care. 2019 Aug 14;9:90. doi: 10.1186/s13613-019-0566-z (PMC6692798; doi:10.1186/s13613-019-0566-z)
Supplement: Supplementary file 7 — Additional file 7: Table S5. Univariate analysis of variables associated with mortality in the propensity score matched cohort. [file 13613_2019_566_MOESM7_ESM.docx]

**Additional Table S5. Univariate analysis of variables associated with mortality in the propensity score matched cohort.**

|  | **Survivors**  **(n=78)** | **Non-survivors**  **(n=30)** | | **P value** | |
| --- | --- | --- | --- | --- | --- |
| Demographic characteristics | | | | |  |
| Age, years | 55 (45-69) | 63 (56-71) | | 0.04 | |
| Gender, male, n (%) | 53 (68%) | 25 (83%) | | 0.17 | |
| Simplified acute physiology score 2 | 33 (24-41) | 44 (36-51) | | <0.001 | |
| Immunocompromised, n (%) | 34 (44%) | 20 (67%) | | 0.053 | |
| Risk factor for acute respiratory failure, n (%) | | | 0.30 | |  |
| Pulmonary | 59 (76%) | 24 (80%) | |  | |
| Extrapulmonary | 10 (13%) | 1 (3.3%) | |  | |
| No risk factor | 9 (12%) | 5 (17%) | |  | |
| Bilateral lung infiltrates, n (%) | 76 (97%) | 30 (100%) | | 0.93 | |
| Under oxygen | | | | |  |
| Glasgow score | 15 (15-15) | 15 (15-15) | | 0.61 | |
| Systolic blood pressure, mm Hg | 132 (116-150) | 126 (118-139) | | 0.25 | |
| Heart rate, per min | 112 (100-126) | 100 (90-110) | | 0.004 | |
| Respiratory rate, per min | 32 (28-38) | 30 (26-39) | | 0.51 | |
| Oxygen flow, l/min | 12 (9-15) | 12 (8-15) | | 0.57 | |
| PaO_2_/FiO_2_, mm Hg | 130 (96-171) | 118 (83-181) | | 0.98 | |
| PaCO_2_, mm Hg | 35 (31-39) | 34 (32-38) | | 0.65 | |
| pH | 7.44 (7.40-7.48) | 7.46 (7.42-7.47) | | 0.94 | |
| Under noninvasive ventilation after 1 hour | | | | |  |
| Respiratory rate, per min | 30 (24-38) | 33 (25-38) | | 0.68 | |
| SpO_2_, % | 98 (96-100) | 98 (95-99) | | 0.60 | |
| Expired tidal volume, mL | 580 (488-724) | 675 (595-762) | | 0.02 | |
| Minute ventilation, L/min | 18.1 (14.6-21.6) | 21.4 (16.5-24.5) | | 0.03 | |
| Pressure support, cm H_2_O | 8 (7-9) | 8 (7-10) | | 0.90 | |
| Positive end-expiratory pressure, cm H_2_O | 5 (5-5) | 5 (5-5) | | 0.06 | |
| FiO_2_, % | 73 (50-100) | 100 (63-100) | | 0.008 | |
| PaO_2_/FiO_2_, mm Hg | 191 (149-266) | 141 (108-223) | | 0.16 | |
| PaO_2_/FiO_2_ < 150 mm Hg, n (%) | 20 (26%) | 15 (50%) | | 0.03 | |
| PaCO_2_, mm Hg | 37 (31-40) | 36 (30-41) | | 0.81 | |
| pH | 7.42 (7.38-7.47) | 7.45 (7.40-7.47) | | 0.83 | |
| Under noninvasive ventilation within the first 24 hours after ICU admission | | | | |  |
| Worst PaO_2_/FiO_2_, mm Hg | 151 (118-219) | 105 (83-129) | | <0.001 | |
| Worst PaO_2_/FiO_2_ < 150 mm Hg, n (%) | 37 (47%) | 25 (83%) | | 0.002 | |
| Acute respiratory distress syndrome, n (%) | 71 (53%) | 30 (100%) | | 0.21 | |
| Outcomes |  |  | |  | |
| Intubation, n (%) | 29 (37%) | 30 (100%) | | <0.001 | |
| ICU length of stay, d | 10 (6-13) | 12 (4-19) | | 0.70 | |
